# On the Synthesis of Graphene Oxide/Titanium Dioxide (GO/TiO_2_) Nanorods and Their Application as Saturable Absorbers for Passive Q-Switched Fiber Lasers

**DOI:** 10.3390/nano14201682

**Published:** 2024-10-20

**Authors:** Zain ul Abedin, Ajaz ul Haq, Rizwan Ahmed, Tahani A. Alrebdi, Ali M. Alshehri, Muhammad Irfan, Haroon Asghar

**Affiliations:** 1 National Centre for Physics, Quaid-i-Azam University Campus, Islamabad 45320, Pakistan; 2 Department of Physics, Mirpur University of Science and Technology, Mirpur 10250, Pakistan; 3 Department of Physics, Hazara University, Mansehra 21300, Pakistan; 4 Department of Physics, College of Science, Princess Nourah Bint Abdulrahman University, P.O. Box 84428, Riyadh 11671, Saudi Arabia; 5 Department of Physics, King Khalid University, P.O. Box 9004, Abha 61413, Saudi Arabia

**Keywords:** pulsed laser sources, synthesis, GO/TiO_2_ nanorods, Q-switched, erbium-doped fiber lasers, saturable-absorber

## Abstract

We report passively Q-switched pulse operation through an erbium-doped fiber laser (EDFL) utilizing graphene oxide/titania (GO/TiO_2_) nanorods as a saturable absorber. The GO/TiO_2_ nanorods were fabricated using a Sol–gel-assisted hydrothermal method. The optical and physical characterization of the GO/TiO_2_ was then characterized using a field-emission-scanning electron microscope (FE-SEM), energy-dispersive X-ray spectroscopy (EDS), and diffuses reflectance spectroscopy (DRS). To investigate the performance of the Q-switched EDFL based on the GO/TiO_2_ SA, the prepared nanorods were mechanically deposited on the fiber ferrule employing adhesion effects of in-dex-matching gel. This integration of the nanorod SA resulted in a self-starting Q-switching opera-tion initiated at a pump power of 17.5 mW and sustained up to 306.9 mW. When the pump range was tuned from 17.5 to 306.9 mW, the emission wavelength varied from 1564.2 to 1562.9 nm, pulse repetition rates increased from 13.87 kHz to 83.33 kHz, and pulse width decreased from 30.27 µs to 3.75 µs. Moreover, at the maximum pump power of 306.9 mW, the laser exhibited an average output power of 0.74 mW, a peak power of 1.54 mW, and a pulse energy of 8.88 nJ. Furthermore, this study investigates the GO/TiO_2_ damage threshold and prolonged stability of the proposed EDFL system.

## 1. Introduction

Pulsed infrared lasers have attracted considerable interest in applications such as laser ranging [1], optical fiber sensing [2], spectral analysis [3], and medical imaging [4]. Possessing the capacity to produce pulses characterized by a narrow bandwidth and elevated energy; pulsed infrared lasers have significant practical value [5]. Typically, pulsed lasers are generated using two methods: mode-locking and Q-switching. Particularly, Q-switched erbium-doped fiber lasers (EDFLs) have garnered considerable interest as a result of their wide array of applications, such as telecommunications, sensing, and medical procedures [6,7,8,9]. Plenty of explorations have been undertaken to innovate various types of Q-switched EDFLs. Due to their elevated output power (milliwatts (mW) to Watts (W)), superior beam quality, cost-efficiency, and versatility in application and design, the use of EDFL has significantly risen in recent years [10,11]. The techniques used for Q-switching can be classified into two primary categories: passive and active. As opposed to actively Q-switched fiber lasers, passively Q-switched variants provide benefits in compactness and design simplicity [12,13]. It is well recognized that a nonlinear saturable absorber (SA) is an essential constituent of passively Q-switched lasers; advancements in SA materials dictate the evolution of passively Q-switched lasers [14]. The SA permits high-intensity light pulses to pass through while absorbing lower-intensity light, effectively modulating the intracavity light and supporting the ultrashort pulse generation. This nonlinear absorption mechanism promotes self-amplitude modulation that stabilizes and compresses the pulses over time. As the pulse propagates through the EDF, it gains energy through stimulated emission, while the SA controls the pulse duration and shape. This process leads to the formation of stable ultrashort pulses, making saturable absorbers essential for generating mode-locked pulses in fiber laser systems. In the race toward new SAs, researchers have increasingly focused on materials exhibiting broader saturable absorption bands (1000 nm or more), enhanced nonlinearity, greater modulation depth, and superior carrier mobility [15]. Previously, semiconductor saturable absorber mirrors (SESAMs) [16,17], carbon nanotubes (CNTs) [12], graphene [18], graphene-like 2D (topological insulators (TIs) [19,20], black phosphorus (BP) [21], MXenes [22], transition metal based dichalcogenides (TMDs) [23,24,25], oxides [26], metal oxides [9,27], layered metal dichalcogenides [11,28] and metal–organic frameworks (MOFs) [29] have functioned as SAs for demonstrating erbium-doped lasers in a passively Q-switched configuration. These materials have arisen as highly attractive choices for many optoelectronic applications owing to their remarkable properties. SESAMs have various benefits that improve the efficiency of ultrafast lasers. Such mirrors are essential for producing higher repetition rates and pulse intensities in passively mode-locked lasers [30]. CNTs are particularly promising because of their short recovery time and wide operating spectrum, plus inexpensive cost and simplicity in production [31]. TIs exhibit various properties, including a tiny band gap in the entire state with a gapless metallic form at the edge/surface [32] due to its distinct characteristics, like its adjustable band gap and wide operating wavelength range, which reveals its suitability as an SA [33]. Transition metal dichalcogenides (TMDs) offer numerous benefits as SAs in optic applications, especially laser technology. Their particular features allow for effective light modulation, making them appropriate for a wide range of optical devices [34]. However, they exhibit certain limitations and present disadvantages. SESAMs are costly and have a smaller absorption bandwidth, being additionally bulky in size [35]. The primary challenges associated with CNTs include poor modulation depth and an absorption efficiency that varies on their diameter [36]. The band gap of TIs is challenging to adjust for photonics applications due to their complexity and lack of clarity [36]. BP is susceptible to laser-induced optical damage, limiting its operating stability. The modulation depth of BP as an SA is quite modest, with reported values ranging from 10.6% to 15%, which may be insufficient for some high-performance applications [37,38]. TMDs’ band gap can be dramatically affected by flaws, resulting in uneven saturable absorption properties. TMDs frequently display complex nonlinear optical feedback, such as nonlinear scattering, which complicates their application as SAs [39]. Numerous metal oxide SAs struggle to maintain stability under changing environmental conditions. Some metal oxides, such as nickel oxide, exhibit strong temperature dependence, which can impact saturation intensity and modulation depth, potentially resulting in performance loss. Certain metal oxides, like zinc oxide, have low saturation intensity, limiting their use in high-power laser setups [40,41,42].

Research into new materials is ongoing to optimize the creation of pulsed lasers, focusing on ensuring their cheap cost, broad absorption, enhanced damage thresholds, and the dependability of their SAs. Concurrently, transition metal oxides (TMOs) have garnered substantial attention [43,44,45,46], and have been suggested as the perfect SA for producing pulsed lasers [45]. Titanium dioxide (TiO_2_) is a transition metal oxide exhibiting rutile, brookite, and anatase crystal forms. TiO_2_ is currently extensively employed in various fields of photonics owing to its exceptional nonlinear optical characteristics. [47,48]. TiO_2_, a metal oxide, is extensively studied for its advantageous features, which include a high refractive index, a broad indirect band gap, significant visible light transmittance, and increased optical nonlinearities [49,50,51]. In addition, TiO_2_’s transparency window spanning from ultraviolet to near-infrared wavelengths makes it an exciting option for several uses, including photocatalysis [52], solar cells [53], and pulse fiber laser technology [43,54]. Despite this fact, TiO_2_ has been effectively employed for pulse production as a light-absorbing material. Researchers have investigated how nanomaterials facilitate the creation of pulsed lasers owing to characteristics of saturable absorption, which provide benefits like affordable, basic manufacturing, and convenient integration [5]. Graphene, widely recognized as a prominent two-dimensional nanomaterial, has emerged as a highly researched topic among optical scientists in recent years [55,56]. Studies are motivated by several unique optical benefits of graphene, including rapid photo-response and ultra-wideband response across the ultraviolet (UV) to terahertz [57]. Despite its notable benefits, graphene has several natural flaws for instance, a weak absorption coefficient of merely 2.3 percent of incoming light per layer for wavelengths from 300 nm to 2500 nm, an absence of a band gap, and vulnerability to damage at even medium intracavity laser intensities [58]. Graphene is now being hybridized with metal oxide nanoparticles to produce a novel binary composite substance that merges the robust mechanical capabilities of the metal oxide alongside the superb optical qualities of graphene, in an attempt to overcome these limitations [59]. Thus, graphene and TiO2 nanoparticles are anticipated to display remarkable qualities with complementing performance and multifunctional ties for various applications. Research carried out [60,61,62] demonstrated that the surface area of the GO/TiO_2_ composite exceeds than bare GO and TiO_2_. Therefore, it can be observed that the integration of GO significantly enhances the particular surface area of the GO/TiO_2_ composite. Researchers proved that the adsorption performances of the GO/TiO_2_ composite were improved compared to those of bare GO and TiO_2_. The combination of graphene oxide and TiO_2_ nanorods is expected to exhibit outstanding properties, providing enhanced efficiency and adaptability for many uses. This method exhibits a similar Q-Switching range and threshold for creating Q-Switched lasers by incorporating SA with prior research. 

In this paper, GO/TiO_2_ nanorods were prepared and utilized as an SA for the demonstration of a passively Q-switched EDFL. The physical appearance and nonlinear absorption characteristics of the GO/TiO_2_ nanorods were investigated experimentally. A passively Q-switched EDFL having a central wavelength of 1564.2 nm was achieved. When the pump power escalated from 17.5 mW to 306.9 mW, the repetition rate varied from 13.87 kHz to 83.33 kHz, the pulse width decreased from 28.27 μs to 3.75 μs, and an average power output of 0.74 mW at the highest available input power of 306.9 mW was achieved. Additionally, by pushing the pump power to its absolute maximum of 306.9 mW, the utmost single-pulse energy of 8.88 nJ and peak power of 1.54 mW were attained. This study shows that GO/TiO_2_-SA could encourage applicants to generate high-power Q-switched pulses.

## 2. Chemicals, Synthesis, and Characterization of Graphene Oxide/Titanium Dioxide (GO/TiO_2_) Nanorods

### 2.1. Chemicals Utilized for GO/TiO_2_ Nanorod Preparation

The standard chemicals of Sigma Aldrich were used here to synthesize nanomaterial for the customizable development of a Q-switched absorber in fiber lasers. Titanium IV isopropoxide-TTIP (C_12_H_28_O_4_Ti) was used for the production of semiconducting inorganic compound TiO_2_, GO powder (2D nanomaterial), ethanol, deionized water, distilled water- (solvents and washing agents), and sodium hydroxide (NaOH); a strong base were used to initiate chemical reaction and the formation of nanoparticles.

### 2.2. Synthesis of Graphene Oxide/Titanium Dioxide (GO/TiO_2_) Nanorods

**Figure 1:**
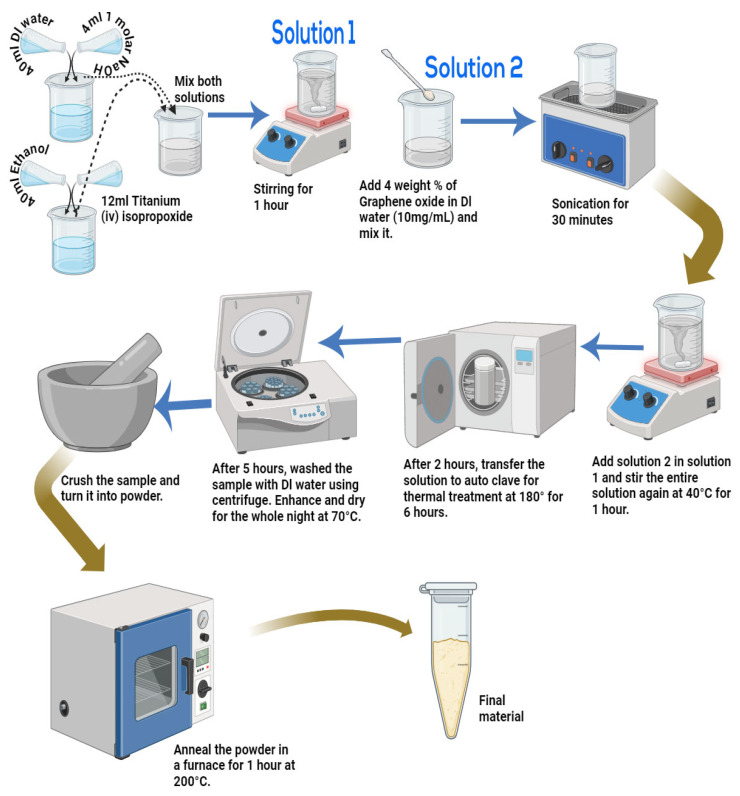
Synthesis diagram of GO/TiO_2_ nanorods using the Sol–gel-assisted hydrothermal method.

The chemical process, the Sol–gel-assisted hydrothermal method, was used to synthesize GO/TiO_2_ nanorods with a ratio of composition of 1:24. For this purpose, 40 mL of deionized water and 4 mL of 1 M NaOH solution were mixed in a beaker. A separate mixture of TTIP of 12 mL measured with a measuring cylinder was mixed in 40 mL of ethanol under mild stirring in another beaker. Once the ingredients were blended, the TTIP solution was included gradually to the first basic solution under continuous stirring for 1 h at room temperature to make a homogeneous mixture. At this stage, the pH was checked, and more drops of the 1 M NaOH aqueous solution were added. Next, the 4 wt% of GO mixed in deionized water (10 mg/mL) was sonicated for 30 min to reduce and homogenize the solution. After sonication, the GO solution was included in the Titania mixture and stirred further for 1 h at 40 °C. Then, the homogeneous solution was formed, where several chemical reactions had been accomplished. The entire homogeneous solution was sealed in an autoclave for hydrothermal treatment, and a temperature of 180 °C was provided inside the microwave oven for 6 h. After the reaction time, the autoclave was cooled down inside the oven at room temperature. The required sample was obtained as a product and washed with distilled water to remove impurities and maintain pH with the help of a centrifuge. The paste was dried at 70 °C and crushed to obtain a fine powder of GO/TiO_2_ nanorods. Then, the powder was annealed at 200 °C for 1 h under air in a muffle furnace to reduce residual stresses and improve the crystalline structure. A schematic diagram for the synthesis of GO/TiO_2_ nanorods using the Sol–gel-assisted hydrothermal method is shown in Figure 1.

### 2.3. Characterizations of GO/TiO_2_Nanorods

The structural properties and crystallite size (D) were determined by X-ray diffractometer D8 Advance Bruker. For optical properties, the reflectance was measured by Perkin Elmer UV-Visible NIR spectrophotometer Lambda 950, and absorbance was taken by UV-Visible absorbance spectrophotometer Lambda 25. The surface morphology, shape, particle size and elemental composition of the samples were examined by electron beam technique–field-emission-scanning electron microscopy (FE-SEM) and energy-dispersive X-ray spectroscopy (EDX) Zeiss Gemini Sigma 500 VP respectively.

#### 2.3.1. XRD Analysis of GO/TiO_2_Nanorods

**Figure 2:**
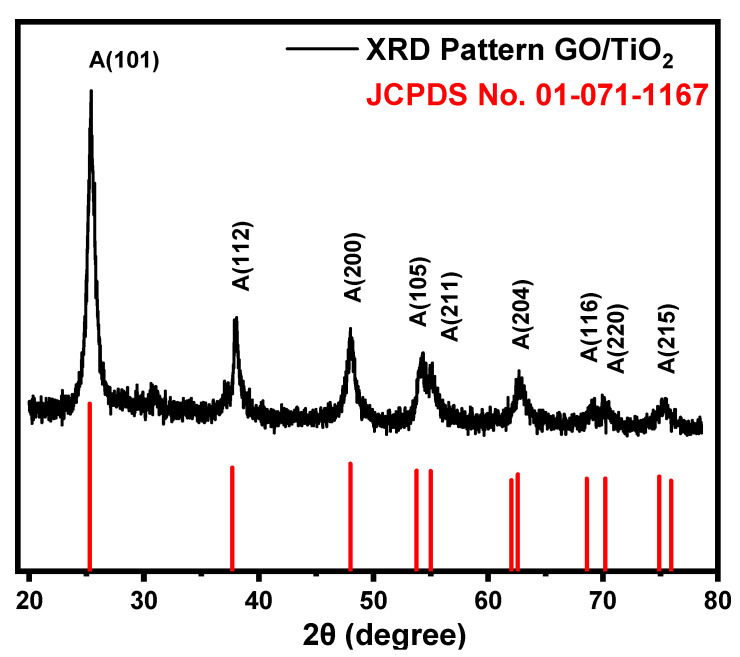
XRD pattern of GO/TiO_2_ nanorods and pattern of JCPDS card no. 01-071-1167 (red).

For structural analysis and confirmation of crystal growth, the XRD pattern of GO/TiO_2_ nanorods was recorded between 2θ values of 20°and 80° with Cu K alpha line (1.5406 Å), 40 kV, and 40 mA. In this analysis, the scan rate was 0.8°/min, while the step size was 0.8°, respectively. Figure 2 shows that the XRD pattern of GO/TiO_2_ nanorods is just like the pure TiO_2_ anatase phase, well matched with JCPDS card no. 01-071-1167. The pattern revealed the tetragonal structure of the sample with lattice constants a = b = 3.7892 Å and c = 9.5770 Å with interfacial angles of α = β = γ = 90°. The diffraction peaks were obtained at 2θ values of 25.4°, 38.08°, 47.98°, 54.34°, 55.08°, 62.68°, 69.02°, 69.94°, and 75.42° in the orientations [101], [112], [200], [105], [211], [204], [116], [220], and [215], respectively. The XRD pattern of GO/TiO_2_ nanorods was satisfied and well matched with pure anatase TiO_2,_ which indicated that there neither the GO peak occurred in the diffraction pattern due to minimum percentage (4 wt% of GO) nor was there new crystal phase formation. The dominant crystal growth occurred at 2θ = 25.4° in the orientation [101]. The crystallite size (D) was determined by using three intense diffraction peaks at 2θ values of 25.4°, 38.08°, and 47.98° with Scherrer’s Equation (1).

(1) $$D=\frac{0.94\;\lambda }{\beta \;cos\;\theta }$$

In the above equation, λ  is the K-α wavelength (1.5406 Å) of the X-ray source, 0.94 is called the shape factor, β is the full width at half maximum (FWHM), which indicates the broadening of the diffraction peak, and θ is the Bragg angle in a degree defined as the conditions of diffraction. The crystallite sizes from the peaks 2θ = 25.4° (β=0.9439), 38.08° (β=0.6352), and 47.98° (β=0.9363) are 54.96 nm, 84.82 nm, and 59.16 nm. The average crystallite is calculated as 66.31 nm.

#### 2.3.2. UV-Visible Diffuse Reflectance Spectroscopy (DRS)

**Figure 3:**
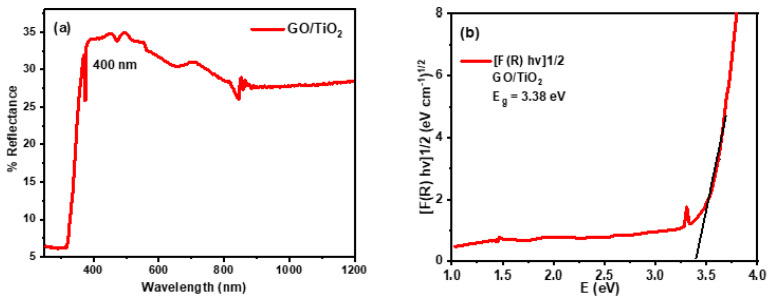
(**a**) Reflectance spectrum of GO/TiO_2_ nanorods and (**b**) band gap energy determined from the Kubelka–Munk function F(R).

UV-visible diffuse reflectance spectroscopy (DRS) was used to investigate optical characteristics like optical reflectance and the band gap energy (E_g_) of synthesized GO/TiO_2_ nanorods, as shown in Figure 3a,b. The UV-visible spectrophotometer was used to record the reflectance spectrum of the specimen within the range of 250 nm to 1200 nm. The maximum reflectance occurred around 400 nm, which is near the visible range. Figure 3a shows that the percentage reflectance became the maximum after 400 nm in the range of incident light. This is because of intrinsic surface properties, band gap, absorption coefficient, particle shape, and size. The absorption of incident light on a material is closely related to the band gap of that material. Therefore, the band gap energy of GO/TiO_2_ nanorods was calculated from DRS data fitted by the Kubelka–Munk function, as shown in Figure 3b. The equation of the Kubelka–Munk function, which is a mathematical function that is implemented to convert reflectance data into absorption vs. band gap energy relationship, is given as

(2) $$F(R_{\infty })=\frac{{\left(1-R_{\infty }\right)}^{2}}{2R}=\frac{k}{s}$$

where $R_{\infty }$ is reflectance, which is the ratio of R_(GO/TiO2)_/R_(reference)_;$\;F(R_{\infty })$ is the Kubelka–Munk function that gives the ratio between the absorption coefficient (k) and scattering coefficient (s). The band gap energy (E_g_) is calculated by

(3) $$k=A{\left(E-E_{\;g}\right)}^{n}$$

A is a constant called absorbance; E is the incident light energy, E_g_ is the band gap energy of the material, and n is the refractive index. For indirect band gap transition (TiO_2_ anatase phase has indirect band gap), n = 1/2. A plot drawn between ${\left[F\left(R\right)hv\right]}^{1/2}$ as a function of E_g_ was used to find the energy band gap of the material. In this instance, the band gap energy of GO/TiO_2_ nanorods determined by the Kubelka–Munk function is 3.38 eV.

#### 2.3.3. Morphology and Elemental Composition of GO/TiO_2_ Nanorods

**Figure 4:**
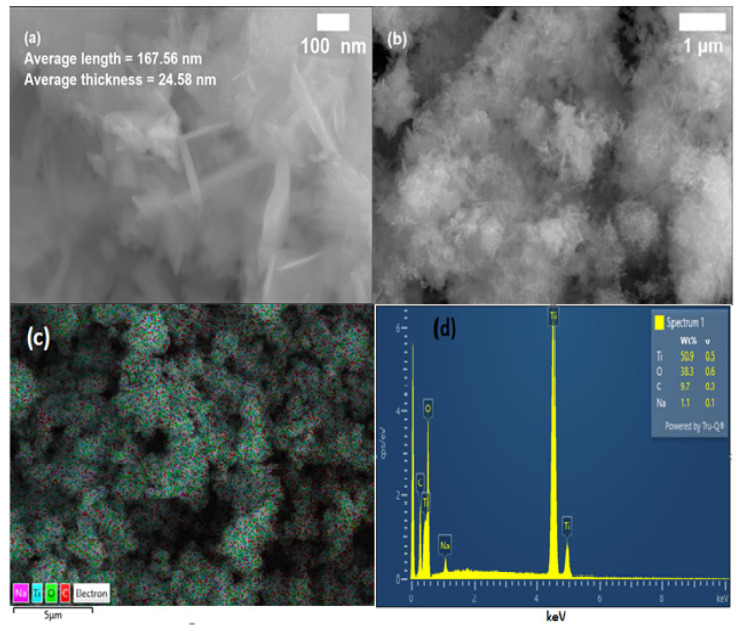
(**a**) FE-SEM high-resolution micrograph of nanorods, (**b**) micrograph with μm scale, (**c**) EDS layered image/elemental mapping, and (**d**) EDX spectrum of the elemental composition of GO/TiO_2_ nanorods.

The combined field-emission-scanning electron microscope (FE-SEM) and energy-dispersive X-ray spectroscopy (EDS) was employed to probe the morphology, shape, and size of synthetic GO/TiO_2_ nanorods and their elemental composition, respectively. Figure 4a shows the FE-SEM micrograph with 100 nm of scale, which reveals that the 1D nanorods were formed during growth and chemical reaction. The 1D nanorods had an average length of 167.56 nm (out of nano-range) with standard deviation (SD = 75.2), and the average thickness of the rods was 24.58 nm with standard deviation (SD = 5.67). The higher SD value for lengths of the nanorods indicates that the formation of the rod was not uniform, and a variety of the lengths were formed. However, the minimum SD values for nanorods thickness indicate that the nanorods were formed of almost the same thickness. In Figure 4b, the micrograph with a 1 μm scale shows the agglomerations of nanorods in the form of bunches, which were accumulated due to weak Van der Waals forces. The EDS layered image/elemental mapping of GO/TiO_2_ nanorods is shown in Figure 4c. From this, it is clear that all the constituent elements (Ti, O, C, and Na) were equally and uniformly distributed during the chemical synthesis of the material. Figure 4d represents the EDS graph of elemental composition, which is a plot between several emitted atomic spectral lines (counts) at a specific energy (keV). This indicates that all the elements that were expected are present in the synthesized material stoichiometrically. The Na was identified due to the use of base sodium hydroxide (NaOH) to initiate the chemical reaction and particle formation. The weight percentage of the constituent elements is given in the table attached with the EDS spectrum.

## 3. Experimental Setup

**Figure 5:**
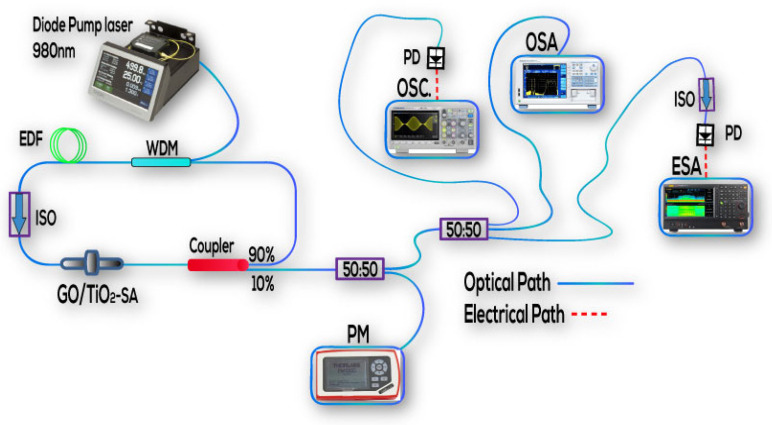
A Q-switched erbium-doped fiber laser’s experimental configurations are based on GO/TiO_2_ nanorods. Abbreviations: WDM: wavelength division multiplexer, EDF: erbium-doped fiber, ISO: optical isolator, ESA: electrical spectrum analyzer, OSA: optical spectrum analyzer, PD: photodiode, OCS: oscilloscope.

The experimental arrangement of a Q-switched EDFL that utilizes GO/TiO_2_ nanorods as an SA is shown in Figure 5. A wavelength division multiplexer (WDM), a continuous wave (CW) pumped diode laser with a center wavelength of 980 nm, was utilized to couple into the EDF. A 2.4 m long EDF with a signal absorption coefficient of 41 dB/m served as the amplification medium. An optical isolator integrated after the doped fiber was used in a fiber-based ring cavity to guarantee a unidirectional flow of the optical signals. For the GO/TiO_2_-SA material to interact with the optical signal, it was positioned in between two ferrules. The EDFL split its output power into two halves at a 90/10 ratio. In total, 90% of the optical signal’s power was returned to the laser ring cavity, and the remaining 10% of the output power was used to examine the characteristics of the EDFL. The optical spectrum was recorded by an optical spectrum analyzer (YOKOGAWA, AQ6370D, Tokyo, Japan) with a minimum resolution of 0.02 nm, covering a wavelength range of 600 to 1700 nm. The RF spectra were obtained using an RF spectrum analyzer (GW INSTEK, GSP-9330, New Taipei City, Taiwan) via a 5 GHz Indium gallium arsenide (InGaAs) photodiode (Thorlabs). A digital oscilloscope (GW INSTEK, GDS-3504, Taiwan) connected to a 5 GHz Indium gallium arsenide (InGaAs) photodiode (Thorlabs) was used to examine the optical pulse train parameters.

## 4. Results and Discussion

**Figure 6:**
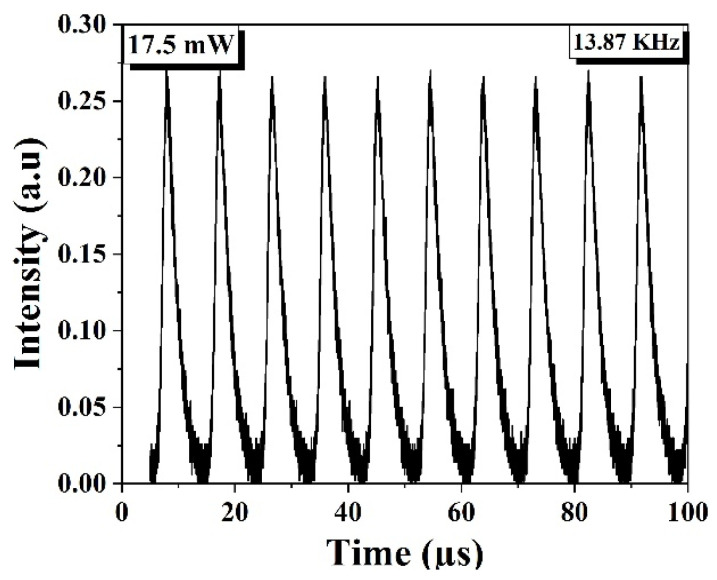
Q-switched pulse operation under 17.5 mW pump power.

The measured results using a proposed experimental arrangement of EDFL show that a Q-switched pulse operation starts under pump power 17.5 mW and remains stable up to 306.9 mW. Figure 6 shows the Q-switched pulse operation under 17.5 mW pump power when the Q-switched operation is triggered.

**Figure 7:**
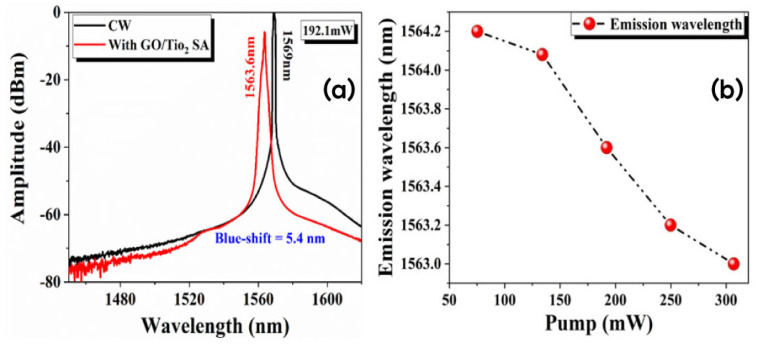
(**a**) Output spectra of the Q-switched EDFL in CW (black line) and pulsed mode (red line). (**b**) Emission wavelength as a function of pump power.

The emission spectrum of the CW-EDFL under 192.1 mW of pump power is shown in Figure 7a, as represented by the black line. The peak wavelength of the emission spectrum is noticed to be 1569 nm. Similarly, under the pump power of 192.1 mW, when the GO/TiO_2_-SA is incorporated inside the laser cavity, the laser emits at 1563.6 nm, as shown in Figure 7a (red line). The comparison of the emission spectra of EDFL subject to GO/TiO_2_-SA and CW mode is recorded under similar pump power. The observed spectra show that a blue shift of 5.4 nm in wavelength occurred due to nonlinear effects, including four-wave-mixing, and self-phase-modulations [63,64,65]. These nonlinear interactions occur with greater pump outputs as light intensity rises in the medium. The process of self-phase modulation involves changing the phase of the propagating light as a result of an intensity dependence on the medium’s refractive index. The blue shift that is seen is caused by this phase shift, which creates additional frequency components that usually widen the spectrum towards shorter wavelengths. Furthermore, thermal effects resulting from increased pump power may alter material characteristics, including fluctuations in refractive index, thus contributing to the wavelength shift. At higher pump power levels, where the light-matter interaction is more intense, these combined effects become more apparent and result in a higher blue shift compared with lower pump powers. The relationship between emission wavelength and pump power is depicted in Figure 7b. The emission wavelength as dependent on pump power denotes the correlation between the input power provided to a gain medium (pump power) and the consequent wavelength of emitted light.

**Figure 8:**
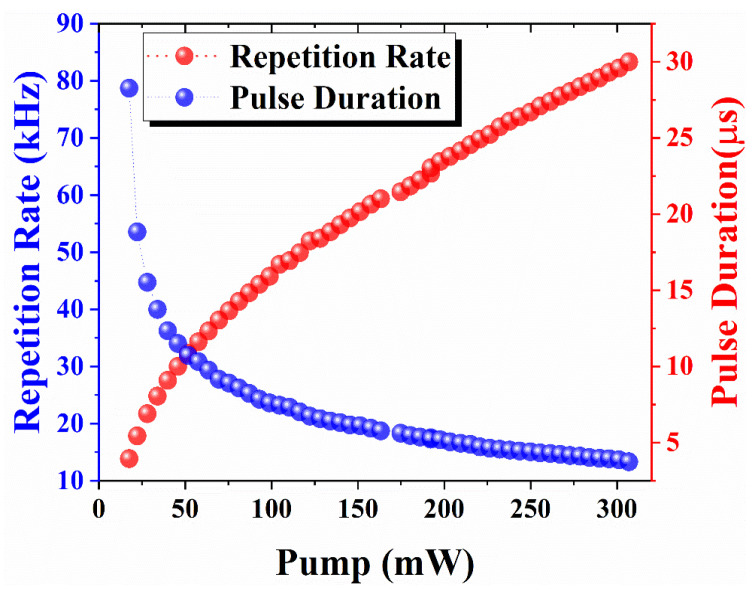
Behavior of pulse repetition rate and pulse duration versus pump power.

Figure 8 depicts the variation in pulse repetition rates and pulse width according to pump power ranges from 17.5 to 306.9 mW. The pulse repetition rate increases from 13.87 to 83.33 kHz, and the pulse duration decreases from 28.27 to 3.75 µs as the pump power increases from 17.5 to 306.9 mW. The rise in pulse repetition rate and decrease in pulse width reveal the Q-switching signature [66]. Additionally the collaboration between pulse width and repetition rate results from the dynamics of the laser cavity and of the gain medium.

**Figure 9:**
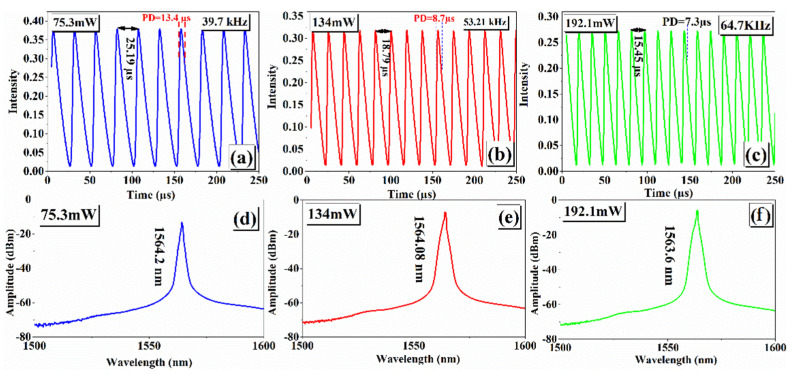
(**a**–**c**) Pulse trace and (**d**–**f**) emission spectra of pulsed EDFL under three chosen pump powers: 75.3, 134, and 192.1 mW.

Figure 9a–c shows the pulse trace of EDFL under three chosen pump powers: 75.3, 134, and 192.1 mW. The measured pulse traces show a pulse interval of 25.19 µs, 18.70 µs, and 15.45 µs corresponds well with the repetition frequencies 39.7 kHz, 53.21 kHz, and 64.7 kHz under pump powers 75.3, 134, and 192.1 mW, respectively. Additionally, under similar pump power, the emission wavelength varied from 1564.2 nm to 1563.6 nm and can be seen in Figure 9d–f. The measured optical emission spectra confirm that a blue shift in the emission wavelength occurred upon tuning the pump power.

**Figure 10:**
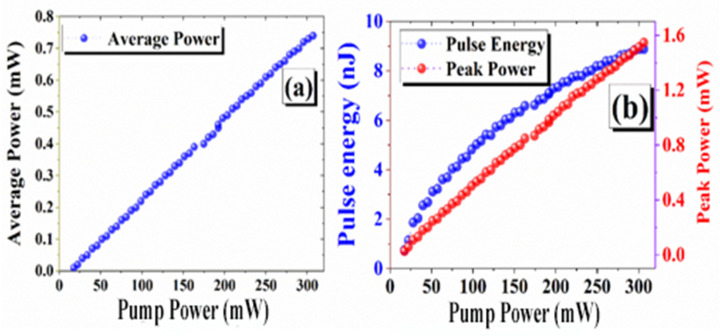
(**a**) Measured average output power and (**b**) pulse energy and peak power as a function of pump power.

In Figure 10a,b, we plotted the average output power corresponding to pulse energy and peak power of the EDFL based on GO/TiO_2_ nanocomposite SA. It is observable that as pump power increases from 17.5 to 306.9 mW, the output power, pulse energy, and peak power increase from 0.01 to 0.74 mW, 0.72 to 8.88 nJ, and 0.02 to 1.54 mW, respectively. In ultrafast laser systems, the linear correlation between average power and peak power with the pump is mostly attributable to the direct proportionality. As pump power rises, energy is delivered into the laser medium, roughly linearly increasing laser output. Peak power specifically correlates with energy per pulse and inversely with pulse width, both of which are affected by pump power. The pulse energy demonstrates sub-linear behavior and saturation resulting from various nonlinear factors in the laser cavity, such as gain saturation and thermal influences. The gain medium becomes saturated upon exceeding the pump power a certain threshold, which means that further rises in pump power do not result in proportionate rises in pulse energy. Moreover, thermal effects may further constrain energy extraction, resulting in pulse energy saturation. There has also been a similar tendency in prior published reports of decreasing pulse energy beyond a particular threshold [24,67].

**Figure 11:**
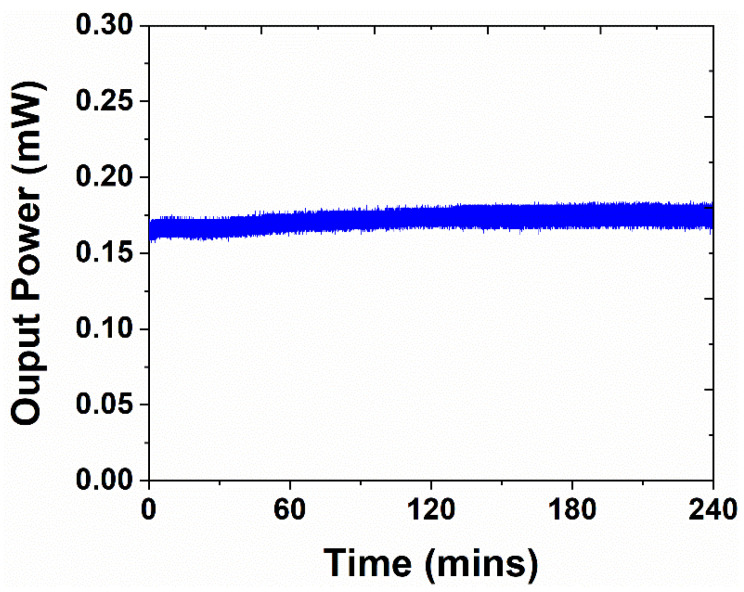
Measured average output power as a function of time to explore the stability of the EDFL system.

The following thoroughly examines the EDFL system’s stability employing GO/TiO_2_ composite as an SA. To evaluate the system stability, the GO/TiO_2_ composite was deposited on the fiber ferrule to behave as an SA, and the pump power was fixed at 75.3 mW. The stability of the system was evaluated in terms of average output power that was continuously monitored and recorded for 4 hrs. The power meter was attached to the computer to continuously record data within 15 min of time intervals. Then, finally, all recorded average output power data were plotted in terms of time (refer to Figure 11). The advantage of this approach is that it measures average output power continuously and offers an excellent approach to measure the stability of the system. The measured results demonstrate that the average pump power remained the same over time, and no fluctuations or instabilities were noticed, indicating the robust stability of the EDFL system.

The important characteristics of pulse performance, including the output power, pulse energy, repetition rate, pulse width, and threshold condition or Q-Switching range of Q-switched operation, are shown in Table 1. It is noteworthy to point out that, afterwards reaching the maximum upper limit of pump power, no physical or performance damage is seen on the GO/TiO_2_ nanocomposite SA. This suggests that the manufactured SA has a damage threshold that is greater than the cavity’s maximum upper capacity of pump power capacity. Table 1 show that this approach has a comparable Q-Switching range and Q-Switching threshold in generating Q-switched lasers by integrating SA with previous work. Low Q-switching thresholds and high damage thresholds have major advantages in terms of improving laser system performance and applications [68,69]. Remarkably, even beyond 306.9 mW, the Q-switching persists, underscoring that the prepared GO/TiO_2_-SA exhibits a damage threshold higher than 306.9 mW. It is noteworthy that the input power was not further elevated beyond this point due to the limitations of the fiber components, which are not designed for operation at much higher powers. This observation suggests that the GO/TiO_2_-basedSA possesses a threshold above 306.9 mW, emphasizing its remarkable characteristics. This threshold delineates the power limits within which reliable Q-switching can be achieved, and this aspect holds considerable significance in the design and operation of EDFLs due to the lowest Q-switching threshold and higher damage threshold that is greater than the maximum pump power present in this cavity to cause a high Q-switching range. Because of the wide Q-switching range in EDFLs, these lasers are more adaptable and perform well in a diverse set of applications. The ability to attain wide Q-switching ranges enables the development of short, high-intensity pulses with adjustable repetition rates and pulse durations [70], which are crucial in applications such as medical treatments, precision material processing, and communication systems [71].

## 5. Conclusions

We experimentally exhibited the potential of a GO/TiO_2_ nanocomposite as a robust nanomaterial saturable absorber, deposited on fiber ferrules using a basic deposition technique. The GO/TiO_2_-SA nanocomposite was prepared using the Sol–gel-assisted hydrothermal method approach, and various characterization methods including FE-SEM, EDS, and DRS were employed to observe the structure and morphology of the GO/TiO_2_. The primary goals of this research were to optimize the fiber laser cavity for improved results and to appraise the performance of GO/TiO_2_ as a saturable absorber within the laser system. Our findings reveal that the fabricated SA exhibits exceptional qualities, enabling stable Q-switched pulses with an emission wavelength of 1564.2 nm. Notably, this fiber laser achieved a maximum repetition rate of 83.33 kHz, underscoring the capability of GO/TiO_2_ to generate rapid optical pulse sequences. Furthermore, we observed minimum pulse duration of 3.75 µs, achieved with a pump power of 306.9 mW, suggesting that GO/TiO_2_ has a damage threshold above 306.9 mW, making it well suited for ultrafast lasers. Under similar pump power conditions, our experimental setup yielded an average output power of 0.74 mW, along with a peak power of 1.54 mW and pulse energy of 8.88 nJ. These results illustrate the effectiveness of the GO/TiO_2_ composite SA in producing pulses powerful enough for a variety of photonics applications. This suggested SA demonstrated better stability during all of our experimental trials. Minimal wavelength changes and regular pulse durations demonstrated its robustness and dependability. These findings underscore the exceptional suitability of GO/TiO_2_ as a promising saturable absorber material for achieving stable Q-switched pulse lasers.

## Figures and Tables

**Table 1:** Comparison of the performance of a GO/TiO_2_ SA-based EDFL with the literature.

SaturableAbsorbers	Integration Method	Technique Used	Pulse Duration (µs)	Repetition Rate (kHz)	Average Power (mW)	Pulse Energy (nJ)	Peak Power (mW)	Q-Switching Range (mW)	Ref.
TiO_2_	Thin Film	Polymer-based thin film	1.84	80.28–120.48	0.148	1.844	0.843	140–240	[28]
TiO_2_	colloid	Doctor Blade Method	2.08	59.84–72.8	2.18	29.91	-	63.41–104.62	[29]
TiO_2_	Thin Film	PVA film	4.12	81.04–90.58	2.05	22.63	-	104.62–145.83	[30]
TiO_2_	Nanoparticles	DC magnetron sputtering technique	0.08	31.8–83.3	0.034	0.13–0.41	0.07–6.80	50.0–270	[31]
GO/TiO_2_	Nanoparticles	Sol–gel method	3.75	83.33	0.74	8.880	1.544	17.5–306.9	This Work

## Data Availability

Data of the results presented in this paper are not publicly available at this time but may be obtained from the authors upon reasonable request.
